# Regulatory mutations in Sin recombinase support a structure-based model of the synaptosome

**DOI:** 10.1111/j.1365-2958.2009.06756.x

**Published:** 2009-06-23

**Authors:** Sally-J Rowland, Martin R Boocock, Arlene L McPherson, Kent W Mouw, Phoebe A Rice, W Marshall Stark

**Affiliations:** 1 University of Glasgow, FBLS, Division of Molecular Genetics, Bower Building, University AvenueGlasgow G12 8QQ, Scotland, UK.; 2 Department of Biochemistry and Molecular Biology, The University of ChicagoChicago, IL 60637, USA.

## Abstract

The resolvase Sin regulates DNA strand exchange by assembling an elaborate interwound synaptosome containing catalytic and regulatory Sin tetramers, and an architectural DNA-bending protein. The crystal structure of the regulatory tetramer was recently solved, providing new insights into the structural basis for regulation. Here we describe the selection and characterization of two classes of Sin mutations that, respectively, bypass or disrupt the functions of the regulatory tetramer. Activating mutations, which allow the catalytic tetramer to assemble and function independently at site I (the crossover site), were found at ∼20% of residues in the N-terminal domain. The most strongly activating mutation (Q115R) stabilized a catalytically active synaptic tetramer *in vitro*. The positions of these mutations suggest that they act by destabilizing the conformation of the ground-state site I-bound dimers, or by stabilizing the altered conformation of the active catalytic tetramer. Mutations that block activation by the regulatory tetramer mapped to just two residues, F52 and R54, supporting a functional role for a previously reported crystallographic dimer–dimer interface. We suggest how F52/R54 contacts between regulatory and catalytic subunits might promote assembly of the active catalytic tetramer within the synaptosome.

## Introduction

**Fig. 1:**
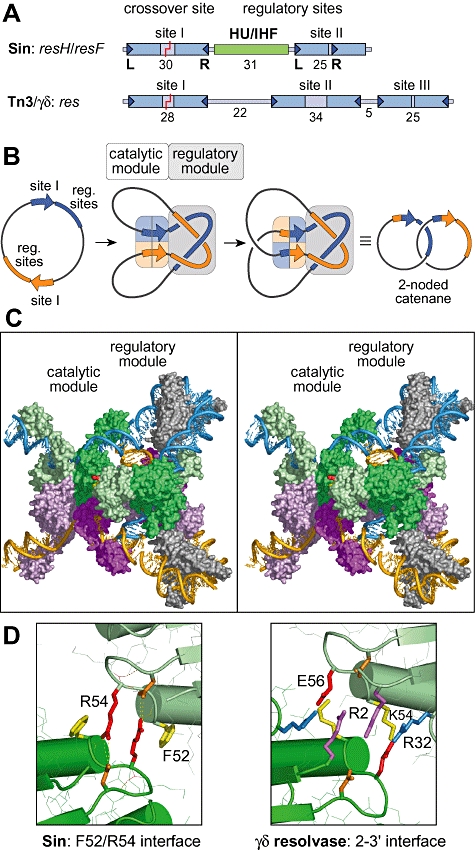
Proposed architecture of the Sin synaptosome. A. Organization of the recombination sites (*res*) in the Sin and Tn3/γδ resolution systems. DNA strand exchange occurs at the centre of the crossover site (site I). In the Sin system, there is only one regulatory binding site for a dimer (site II), compared with two in the Tn3/γδ system (sites II and III). The second regulatory site in the Sin system is for an architectural protein (HU in *resH*, IHF in *resF*). The lengths of the recombinase binding sites, and of the site I–site II spacers, are given (bp). B. Topology of the synaptosome and of the recombination reactions catalysed by Sin and Tn3/γδ resolvase. The regulatory module is formed by synapsis of the regulatory sites; the catalytic module is formed by synapsis of the crossover sites. Since the synaptosome traps three negative supercoils, and strand exchange has a right-handed sense, the resolution product is a specific two-noded catenane. C. Stereo pair showing a model of the Sin synaptosome assembled by rigid-body docking of available crystal structures for Sin (pdb: 2R0Q; Mouw *et al*., 2008), γδ resolvase (pdb: 1ZR4; Li *et al*., 2005) and IHF (pdb: 1IHF; Rice *et al.*, 1996) (Mouw *et al*., 2008). The two *resF* sites are shown in orange and blue, and the recombinase subunits bound to these sites are in shades of violet/magenta and green respectively; the IHF heterodimers are grey. Residues F52 (yellow) and R54 (red) are visible in the regulatory Sin dimer bound at the blue *resF* site. The catalytic and regulatory tetramers are in close proximity and could make contact, but the interface would be asymmetric (see text). See Fig. 9F for a 90° rotated view of this model, and Fig. 9E for a modified model incorporating conformational adjustments that allow the catalytic and regulatory tetramers to make contact using the pseudo-symmetric F52/R54 interface. D. Subunit interfaces thought to be involved in contacts between the regulatory module and the catalytic module in the Sin and Tn3/γδ resolvase systems. The F52/R54 dimer–dimer interface of Sin (F52, yellow; R54, red; D57, orange) is compared with the 2–3′ interface of γδ resolvase (R2, magenta; R32, blue; K54, yellow; E56, red; D59, orange). Residues at equivalent positions in the secondary structure (see Fig. S1) have the same colour. The F52/R54 interface has a cation/π stack, whereas the 2–3′ interface has a stack of four arginine side-chains.

Sin is a resolvase of the serine recombinase family that is encoded by the *Staphylococcus aureus* multiresistance plasmid pI9789 (Rowland *et al*., 2002); it has many similarities to the archetypal resolvases of Tn*3*, γδ and Tn*21* (Grindley, 2002; Grindley *et al*, 2006). Serine recombinases have diverse biological functions as resolvases, DNA invertases, transposases and integrases, and they have many potential applications in biotechnology and gene therapy (Akopian and Stark, 2005; Calos, 2006). They are also model systems for studying how catalytic events at two or more DNA sites are coordinated to achieve a precise outcome (Krasnow and Cozzarelli, 1983; Li *et al*., 2005). Regulation of catalysis is a critical issue for all serine recombinases, because the catalytic tetramer makes transient DNA double-strand breaks (DSBs) in the two synapsed crossover sites, and uses a potentially hazardous rotational mechanism for strand exchange (Stark *et al*., 1989; Boocock *et al*., 1995; Grindley, 2002; Dhar *et al*., 2004; Li *et al*., 2005). In the Sin and Tn3/γδ resolution systems, separate regulatory sites control the choice and alignment of two crossover sites, driving the resolution reaction in the forward direction (Fig. 1A and B) (Arnold *et al*., 1999; Rowland *et al*., 2005).

**Fig. 9:**
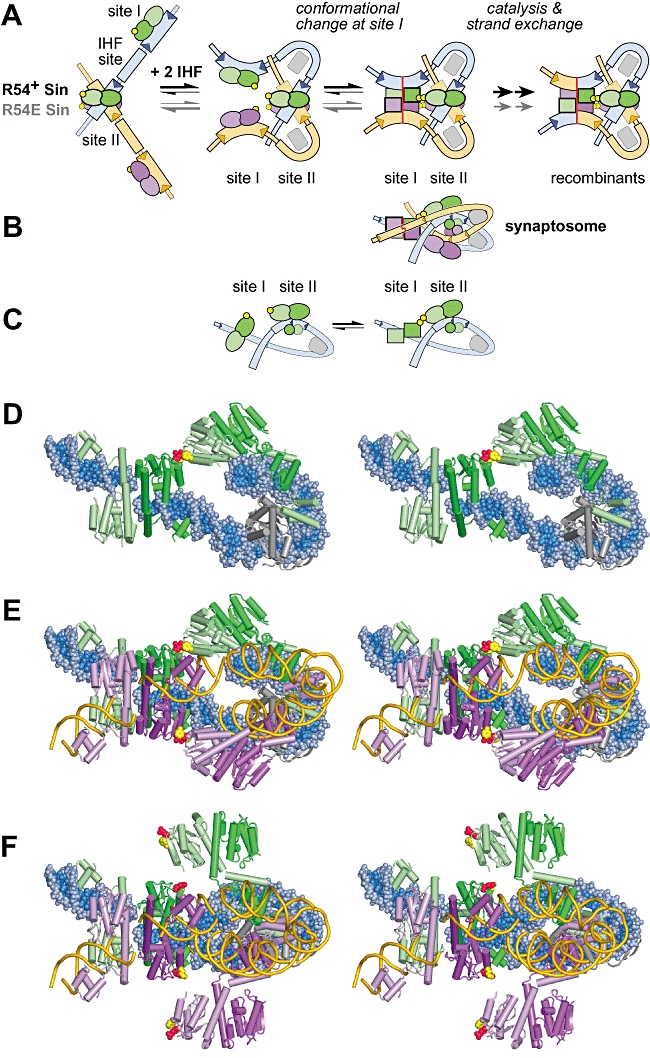
Proposed mechanism for cooperation between the F52/R54 interface and IHF in assembly of the site I tetramer within the synaptosome. The molecular models in (D), (E) and (F) are shown as stereo pairs. A. Proposed pathway for assembly of the synaptosome. Sin dimers in the ‘closed’ conformation are shown as ovals; dimers in the ‘open’ conformation, as in the site I synaptic tetramer, are shown as squares. Residues that form the F52/R54 interface are represented by yellow circles, and IHF is represented by a grey rectangle. B. The synaptosome in (A) viewed from below (i.e. rotated 90°). This is a cartoon representation of the molecular model in (E). C. Proposed looping interaction, mediated by the F52/R54 interface, between Sin dimers bound at sites I and II within the same *resF* site, shown out of the context of the synaptosome for clarity. Each cartoon is half of the model shown above it in (A), viewed from below (as in B). We suggest that the looping interaction stabilizes an ‘open’ conformation of the dimer at site I (squares) relative to the ‘closed’ conformation (ovals). A molecular model of the right-hand cartoon is shown in (D). D. Molecular model of the proposed looping interaction; this is one-half of the complete synaptosome model shown in (E). Two Sin dimers bound at the same *resF* site interact through the pseudo-symmetric F52/R54 interface as seen in the Sin site II crystal structure (pdb: 2R0Q). The site I dimer is in an ‘open’ conformation, as shown in Fig. 8B. The DNA is in spacefill, and the Sin dimers (pale/dark green) and IHF heterodimers (grey) are in cartoon representation; the side-chains of Sin residues F52 (yellow) and R54 (red) are highlighted in spacefill. E. Molecular model of the synaptosome incorporating the F52/R54 interface (shown in cartoon form in B). This model is essentially equivalent to fig. 7C of Mouw *et al*. (2008), except that all four catalytic domains in the site I synaptic tetramer were modelled by fitting segments of the Sin structure onto the γδ resolvase co-ordinates (cf. Fig. 8C here). The modelled synaptosome can be viewed as comprising two identical *resF* loops, held together by NTD interactions at site I, and CTD interactions at site II. The ‘back’ loop is as shown in (D); in the ‘front’ loop, the Sin dimers (lilac/purple) and the DNA (gold) are in cartoon representation (the IHF is omitted for clarity). Sin subunits at sites I and II make contact through the crystallographic F52/R54 interface, highlighted in spacefill (F52, yellow; R54, red). In order to create this interface, a conformational adjustment was made to the crystallographic site II tetramer: the NTDs were rotated towards the site I tetramer by straightening the kink in the E helix (of the pale green and lilac subunits). The positions of the regulatory site DNAs (relative to the site I DNAs) are slightly different from those shown in (F). F. The original molecular model of the synaptosome made by rigid body docking of known crystal structures, exactly as shown as in fig. 7A and B of Mouw *et al*. (2008) except that the IHF in the ‘front’ loop has been omitted for clarity. Note that residues F52 (yellow) and R54 (red) in the Sin site II tetramer are distant from their potential partners in the site I tetramer. The site I tetramer here is the γδ resolvase structure (and not the modelled Sin tetramer shown in E). γδ resolvase residues K54 (yellow) and E56 (red), which correspond to F52 and R54 in Sin, are highlighted (see Fig. 1D and Fig. S1). The particular conformation of the Sin NTDs seen in the site II tetramer may result from crystal packing forces.

Recombination by wild-type (WT) Sin requires an elaborate protein/DNA complex called the synaptosome, in which two 86 bp recombination sites, *resH*, are interwound to trap three negative supercoils (Fig. 1A and B). The synaptosome comprises the catalytic tetramer bound to the two crossover sites (site I) and a separate regulatory module, in which two regulatory sites (site II) are synapsed by a Sin tetramer, and the site I–site II spacers are bent by an architectural protein, HU (Rowland *et al*., 2002; 2005). HU can be functionally replaced by the sequence-specific DNA-bending protein IHF, if a cognate binding site is placed at the centre of the site I–site II spacer (Fig. 1A); the modified recombination site is called *resF* (Rowland *et al*., 2006). The *resH*/*resF* site is far more compact than the Tn3/γδ*res* site, which has a second regulatory resolvase binding site instead of the HU/IHF site (Fig. 1A).

Like other resolvases, Sin acts selectively on recombination sites that are arranged in direct repeat in a supercoiled DNA circle (Fig. 1B) (Rowland *et al*., 2002). Selectivity arises because recombination is contingent on assembly of the synaptosome, and thereby on synapsis of the regulatory sites (Rowland *et al*., 2005; Mouw *et al*., 2008), and this requires a substrate with the appropriate topology (Stark and Boocock, 1995). The regulatory sites thus promote resolution, and block undesirable competing reactions such as inversion.

The protein–DNA architecture of resolvase synaptosomes has been the focus of considerable research and conjecture (Rice and Steitz, 1994; Murley and Grindley, 1998; Sarkis *et al*., 2001; Rowland *et al*., 2006). A recent crystal structure of the Sin regulatory tetramer at site II revealed an unexpected synapsis interface between the small C-terminal DNA-binding domains (CTDs), and genetic data established that this interface is needed in the functional synaptosome (Mouw *et al*., 2008). The new structure enabled us to assemble a model of the complete Sin synaptosome (Fig. 1C; Mouw *et al*., 2008), by rigid-body docking of available structures for Sin (site II synapse), γδ resolvase (site I synapse) and IHF, constrained by the known arrangement of the three binding sites within *resF* (Rowland *et al*., 2006)*.* This model successfully accounts for the interwound topology of the Sin synaptosome. The Sin regulatory tetramer has a ‘DNA-in’ configuration that contrasts with the ‘DNA-out’ configuration of the catalytic tetramer (Nöllmann *et al*., 2004; Li *et al*., 2005), and with the ‘DNA-out’ configuration suggested for regulatory tetramers in previous published models of the Tn3/γδ resolvase and Sin synaptosomes (Murley and Grindley, 1998; Sarkis *et al*., 2001; Rowland *et al*., 2002). Our new structure-based model of the Sin synaptosome thus places the large domains of the regulatory subunits bound at site II on the outside of the complex, in positions where they could readily contact two large domains of the catalytic tetramer from opposite sides (Fig. 1C; see Fig. 9F for a rotated view), whereas previous models of the Tn3/γδ resolvase and Sin synaptosomes placed the large domains of the regulatory subunits in contact with each other on the inside of the complex.

It has been proposed that specific contacts between the regulatory and catalytic subunits within the synaptosome are needed to activate recombination at site I (Murley and Grindley, 1998; Sarkis *et al*., 2001; Mouw *et al*., 2008). We previously reported that a directed mutation at Sin residue R54 can disrupt regulation without affecting synapsis of the regulatory sites (Mouw *et al*., 2008). The rigid-body docking model of the Sin synaptosome (Fig. 1C) predicts a contact interface that is asymmetric, involving side-chains around residues R54 and I40 in catalytic and regulatory subunits respectively. However, by making conformational adjustments to the regulatory subunits in the model, the contact interface can be modelled using the pseudo-symmetric ‘F52/R54′ dimer–dimer packing interface seen in the Sin crystals, in which the side-chains of residues F52 and R54 from two subunits are interdigitated (Fig. 1D; Mouw *et al*., 2008). It was therefore important to investigate the potential role of the F52/R54 interface. An intriguing feature of this interface is its resemblance to the 2–3′ regulatory interface of Tn3/γδ resolvase (Fig. 1D) (Salvo and Grindley, 1988; Hughes *et al*., 1990; Murley and Grindley, 1998). In the Tn3/γδ system, the 2–3′ interface is essential for recombination, and is important for resolvase-mediated looping within one *res* site and for synapsis of the regulatory sites (Hughes *et al*., 1990; Murley and Grindley, 1998).

The mechanism whereby the regulatory module stimulates recombination at site I is not known. Synapsis at site I involves dramatic conformational rearrangements in the recombinase, as illustrated by crystal structures of γδ resolvase in an ‘inactive’ pre-synaptic dimer, and in an ‘active’ post-cleavage synaptic tetramer (Yang and Steitz, 1995; Li *et al*., 2005). The intermediate steps in site I synapsis and DNA strand cleavage are less well understood (Nöllmann *et al*., 2004; Kamtekar *et al*., 2006). Recombination can occur at site I in the absence of the regulatory module, but this requires ‘activating’ mutations in the enzyme (Arnold *et al*., 1999; Burke *et al*., 2004; Rowland *et al*., 2005). For Tn3/γδ resolvase, multiple activating mutations are necessary for assembly of a stable site I synaptic tetramer (Sarkis *et al*., 2001; Nöllmann *et al*., 2004), and this stabilization is the likely basis for the activation of recombination (Olorunniji *et al*., 2008). We previously identified one activating mutation in Sin (I100T) that supports site I × site I recombination (Rowland *et al*., 2005). However, it is not yet clear whether the main function of the regulatory module in the WT systems is to control site I synapsis or later reaction steps.

Our aim in this study was to define protein–protein interfaces that are critical for assembly of the synaptosome and regulation of recombination. We describe two distinct classes of regulatory mutations in Sin. We show that activating mutations map to diverse positions in the N-terminal catalytic domain, and can stabilize a catalytically active site I synaptic tetramer, and we suggest how they might have this effect. We also show that mutations that block activation by the regulatory module map exclusively to residues F52 and R54, demonstrating the functional relevance of the crystallographic F52/R54 interface. The data support a model of the Sin synaptosome that incorporates this interface (Mouw *et al*., 2008), and we suggest a mechanism for the regulation of catalysis that may be applicable to other serine recombinases.

## Results

### Selection and characterization of activating mutations in Sin

**Fig. 2:**
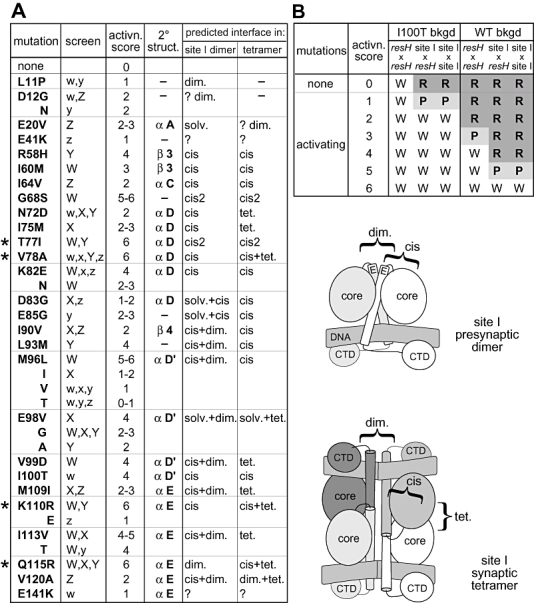
Activating mutations in Sin. A. The activating mutations listed were selected from libraries of random mutants generated in three different backgrounds (see text for details): WT (screen W), I100T (screen X) and R54E/I100T (screens Y and Z). Many mutations were selected in more than one background, as indicated: upper case (W, X, Y, Z) denotes mutations selected without secondary mutations; lower case (w, x, y, z) denotes mutations selected along with secondary mutations, but shown to confer a phenotype after separation. The ‘activation score’ is a measure of how strongly a mutation activates recombination (see B). The location of each mutation in the secondary structure of Sin bound at site II (Mouw *et al*., 2008) is given. Structural interfaces on which mutated residues are predicted to reside in the inactive site I dimer, and in the active site I synaptic tetramer, are also listed (‘predicted interface in site I dimer/tetramer’). Interfaces are defined in the accompanying cartoons, which subdivide each subunit into the ‘core’ catalytic domains (containing helices A, B, C, D and D′) the E helix, and the CTD: dim., interfaces between two subunits in the same dimer; cis, interfaces between helix E and the core domain of the same subunit, or between helix D′ and the remainder of the core domain of the same subunit; cis2, interfaces between helix C and helix D within the core domain; tet., the synapsis interface between the two dimers in the tetramer; ‘?’ denotes residues that are disordered in the Sin structure (residue E41) or whose structural role is unclear. The assignments are based on the Sin site II-dimer structure (Fig. 8A; Mouw *et al*., 2008), on the γδ resolvase site I synaptic tetramer structure (Li *et al*., 2005), and on a Sin site I synaptic tetramer generated by homology modelling (Figs 1C and 8C). B. The ‘activation score’ of a mutation is a convenient numerical measure of the extent to which the mutation activates recombination. Each score represents a specific set of *in vivo* phenotypes conferred by the mutation in WT and I100T Sin backgrounds, in assays with *resH* ×*resH*, *resH* × site I and site I × site I substrates. W (white colonies) indicates complete resolution; P (pink colonies) indicates partial resolution; R (red colonies) indicates no (or very slow) resolution. The WT and I100T Sin backgrounds provide different ‘windows’ of sensitivity. For example, weak activating mutations such as N72D affect the phenotype in the I100T background, but not in the WT background. The maximum score of ‘6’[given to four mutations, indicated by asterisk (*) in (A)] indicates complete resolution of all three substrates in the WT background.

**Fig. 3:**
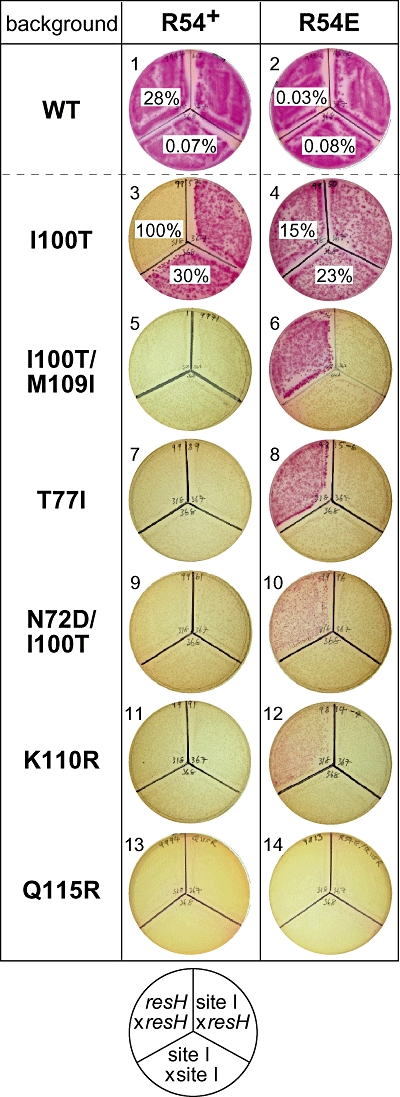
The mutation R54E selectively inhibits *resH* × *resH* recombination *in vivo.* The assays show how R54E affects recombination of *resH* × *resH*, *resH* × site I and site I × site I substrates by WT Sin, various activated mutants. White colonies indicate efficient recombination; red colonies indicate slow, or no, recombination. Selective inhibition of *resH* × *resH* recombination by R54E is evident in the I100T/M109I, T77I, N72D/I100T and K110R backgrounds (red or pink colonies). For *resH* × *resH* and site I × site I recombination in plates 1–4, the percentage of recombined substrate is given after pooled colonies were grown in liquid culture for ∼60 generations. [The value 100% (plate 3) therefore cannot be compared with the other values when estimating relative rates, because recombination has gone to completion.] With the exception of I100T, all of the R54^+^ single and double mutants shown have an ‘activation score’ of 6 (Fig. 2B), although they may not all be activated to the same extent (recombination has gone to completion).

An activating mutation is defined here as any mutation that promotes recombination of site I × site I substrates, bypassing the requirement for the regulatory sites; we previously reported one such mutation in Sin (I100T; Rowland *et al*., 2005). We have now identified activating mutations at an additional 26 residues, using four different criteria to screen large libraries of mutants with random changes in the entire Sin reading frame (screens W, X, Y and Z; Fig. 2A) (see below). To select mutants, we used an *in vivo* recombination assay in which a substrate plasmid, containing a reporter gene (*galK*) flanked by directly repeated copies of *resH* or site I, is complemented with a Sin-expressing plasmid (Rowland *et al*., 2005). On indicator plates, white colonies are seen if resolution (deletion of *galK*) is efficient, while red colonies are seen if resolution is slow or absent; WT Sin recombines a *resH* × *resH* substrate slowly, giving red colonies (e.g. see Fig. 3, plate 1).

**Fig. 8:**
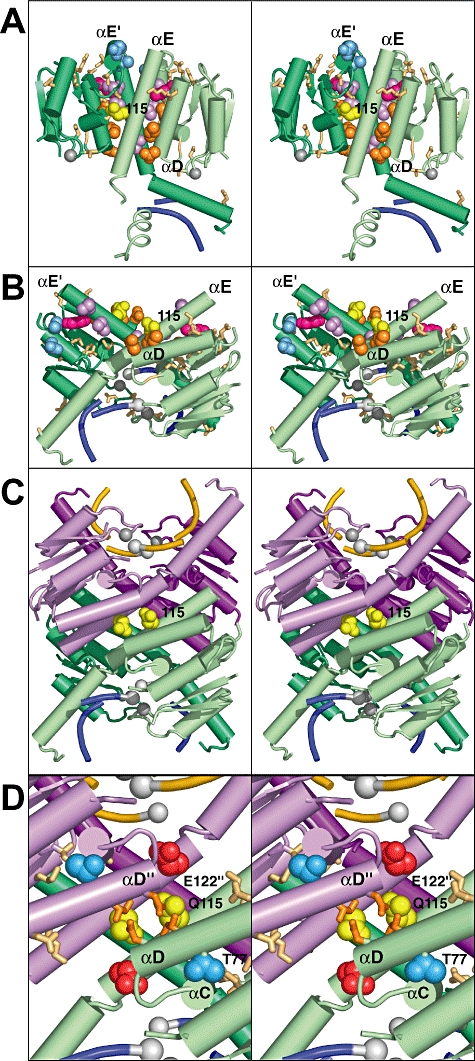
Activating mutations in Sin are located on structural interfaces likely to be involved in assembly of the site I synaptic tetramer. All structures are shown as stereo pairs. A. Crystal structure of the WT Sin dimer bound at site II of *resH* (pdb: 2R0Q; Mouw *et al*., 2008). The CTDs (residues 148–202) are omitted. The WT dimer is thought to bind in a similar ‘closed’ conformation at site I, as seen in the crystal structure of WT γδ resolvase bound at site I (pdb: 1GDT; Yang and Steitz, 1995). Side-chains for all residues with activating mutations (Fig. 2A) are highlighted as sticks (light brown) or in spacefill: N72, I75 and V78 (orange; D helix); E98 and V99 (blue; D′ helix), M109, I113 and V120 (violet; E helix); K110 (pink; E helix); Q115 (yellow; E helix). Note that most of the 10 residues shown in spacefill are largely buried in this ‘closed’ dimer, whereas all are exposed on the top surface of the hypothetical ‘open’ dimer shown in (B), i.e. they are at the dimer–dimer interface of the modelled site I synaptic tetramer shown in (C) (the ‘tet’ interface in the last column of Fig. 2A). Grey spheres mark the position of the serine nucleophile (S9) in each subunit. Only 4 bp of the DNA backbone near the centre of site II are shown (blue). B. Hypothetical model of a Sin dimer bound at site I in an ‘open’ conformation, equivalent to one-half of the modelled Sin site I synaptic tetramer shown in (C). Positions of activating mutations are highlighted, as in (A). C. Hypothetical model of a Sin site I synaptic tetramer (the CTDs are not shown). This model is based on the crystal structure of the γδ resolvase site I synaptic tetramer (pdb: 1ZR4): residues 1–119 of each subunit in the γδ resolvase tetramer were replaced by segments of the Sin crystal structure (2R0Q, chain D). Residues 1–102 (core domain) and 103–125 (helix E) of Sin were treated as independent rigid bodies, and fitted to equivalent Cα positions in the γδ resolvase tetramer. The remainder of the γδ resolvase structure (residues 120–183 and the site I DNA) was not altered. Note that the aim was simply to model the likely environment of residues in the Sin synaptic tetramer, including ∼10 Sin residues (96–100 in helix D′ and 103–107 at the N-terminus of helix E) that have no direct equivalent in the γδ structure; no attempt was made to model side-chain conformations. Residue Q115 is highlighted in spacefill (yellow). Only the central 8 bp of site I DNA (blue or yellow backbone, containing a resolvase-induced DSB) are shown; white spheres represent DNA phosphates covalently joined to the serine nucleophile (S10) in 1ZR4. D. Close-up view of Q115 and nearby residues in the modelled Sin site I synaptic tetramer shown in (C). Side-chains are highlighted as in (A) and (B) [except that N72 and I75 are shown as sticks (orange) instead of spacefill]; also shown are E122 (red) and T77 (blue).

The first screen (screen W; Fig. 2A) selected mutants that can recombine a *resH* × *resH* substrate more efficiently than WT Sin (i.e. giving white or pink colonies); this was the criterion used previously to select I100T Sin (Rowland *et al*., 2005). With one exception (see below), the mutations that were selected on this basis also promoted recombination of a site I × site I substrate. The *resH* × *resH* screen therefore primarily identifies activating mutations that directly promote recombination at site I, rather than mutations that act indirectly through the regulatory sites. Note that the low rate of *resH* × *resH* recombination by WT Sin *in vivo* may be due to weak activation by the regulatory sites, perhaps because the available *Escherichia coli* DNA-bending protein(s) do not function optimally (Rowland *et al*., 2005).

In the second selection procedure (screen X; Fig. 2A), we looked for mutations that increase the efficiency of site I × site I recombination by I100T Sin. This was possible because I100T Sin recombines the site I × site I substrate slowly *in vivo*, giving red colonies (Rowland *et al*., 2005; Fig. 3, plate 3); we could therefore select mutants that give white or pink colonies.

The activating mutations selected in screens W and X are listed in Fig. 2A, together with activating mutations selected in two other screens (Y and Z; see next section). Nearly half of the mutations were selected in more than one screen. All are in the N-terminal catalytic domain of Sin, and at six residues two or more different activating changes were identified. An ‘activation score’ was assigned for each mutation (from 1 to 6; Fig. 2A), based on how it affects recombination of *resH* × *resH*, *resH* × site I and site I × site I substrates *in vivo*, in WT and/or I100T backgrounds (Fig. 2B). All of the mutations in Fig. 2A were shown to promote site I × site I recombination in a WT or I100T Sin background, either in the indicator plate assay or by plasmid DNA analysis (data not shown). With most of the activating mutations, recombination was nevertheless further stimulated by the regulatory sites when they were present in both partners (i.e. the *resH* × *resH* substrate; Fig. 2B). Only four single mutations (T77I, V78A, K110R and Q115R) gave the maximum detectable level of activity with the site I × site I substrate in a WT background (i.e. white colonies, e.g. Fig. 3, plates 7, 11 and 13).

In screen W, we selected I60M Sin (Fig. 2A) and also I60M/H166R Sin, and found that the double mutant recombines a *resH* × *resH* substrate more efficiently than the single mutant (data not shown). However, H166R (unlike I60M) does not promote recombination of a site I × site I substrate (data not shown). Interestingly, H166R was also selected in a screen for mutations that rescue *resH* × *resH* recombination by a CTD mutant defective in site II synapsis; H166 is located close to the CTD synapsis interface and the mutation probably stabilizes this interface (Mouw *et al*., 2008). In summary, H166R is the only mutation identified here that stimulates *resH* × *resH* recombination by enhancing regulatory site functions, rather than by acting directly on site I functions; its effect is small compared with the ‘classical’ activating mutations listed in Fig. 2A.

### Activating mutations can rescue the recombination defect conferred by R54E

**Fig. 7:**
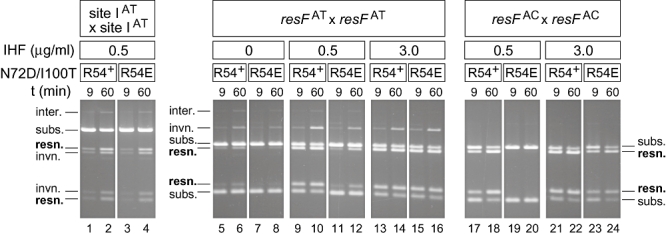
An F52/R54 interface mutation (R54E) inhibits recombination *in vitro* when site II is present and the IHF concentration is low. Recombination of supercoiled site I^AT^, *resF*^AT^ and *resF*^AC^ substrates (∼8 nM) by the activated mutant N72D/I100T (∼250 nM) and its derivative R54E/N72D/I100T (∼300 nM), in the presence of 0, 0.5 or 3.0 μg ml^−1^ IHF (0, 23 or 136 nM), as indicated. Reaction samples taken at 9 and 60 min were digested with XhoI and analysed on a 1% agarose gel. The site I^AT^ and *resF*^AT^/*resF*^AC^ substrates give substrate (subs.) and product (resn., resolution; invn., inversion) fragments of different sizes. The *resF*^AT^ and *resF*^AC^ sites are identical apart from the dinucleotide at the centre of site I; they were referred to previously as *resF*^E^ and *resF*^D^ respectively (Rowland *et al*., 2005). Note that the concentration of free IHF will be lower than expected, because IHF binds with high affinity to vector sequences in the supercoiled DNA substrate (data not shown; Prentki *et al*., 1987).

We previously reported that residue R54 of Sin is important for proper functioning of the regulatory tetramer assembled at site II (Mouw *et al*., 2008). A designed mutation, R54E, strongly inhibits *resH* × *resH* recombination *in vivo* by the activated mutant T77I Sin, without affecting the site I × site I recombination phenotype (see Fig. 3, plates 7 and 8; R54E has little effect on site I × site I recombination *in vitro*, e.g. as shown in Fig. 7, lanes 1–4). The inhibitory effect requires the CTD synapsis interface and site II in both recombination sites, suggesting that assembly of an R54E regulatory tetramer at site II can inhibit, rather than stimulate, recombination at site I (Mouw *et al*., 2008). Here we test whether R54E can selectively inhibit *resH* × *resH* recombination by other activated mutants, and by WT Sin.

To determine how the R54E mutation affects WT Sin and the moderately activated mutant I100T, recombination was quantified by analysing the plasmid DNA after ∼60 generations of cell growth. R54E inhibited *resH* × *resH* recombination by WT Sin by ∼1000-fold (Fig. 3, plates 1 and 2). R54E inhibited *resH* × *resH* recombination by I100T Sin by over 6-fold (Fig. 3, plates 3 and 4), but inhibited site I × site I recombination only modestly (suggesting little direct effect on the catalytic tetramer). As indicated by the colony colours in Fig. 3 (plates 5–12), R54E inhibited *resH* × *resH* recombination, but not *resH* × site I or site I × site I recombination, by the highly activated mutants I100T/M109I, N72D/I100T and K110R. However, recombination by Q115R Sin (also highly activated) appeared to be unaffected by R54E (Fig. 3, plates 11 and 12). In summary, R54E selectively inhibited *resH* × *resH* recombination in several different activated mutant backgrounds, reducing it to a level below that of site I × site I recombination. However, with the single mutant Q115R, and with combinations of activating mutations (e.g. T77I/I100T; data not shown), efficient *resH* × *resH* recombination persisted in the presence of R54E. This suggests that the inhibitory effect of R54E can be overcome by strongly activating mutations, most effectively by Q115R.

To identify mutations that can suppress the inhibitory effect of R54E on *resH* × *resH* recombination, we looked for mutations that completely or partially rescue recombination by the double mutant R54E/I100T (i.e. that result in white or pink colonies with the *resH* × *resH* substrate; see Fig. 3, plates 3 and 4). All of the mutations selected in this screen were found to be activating mutations (screen Y; Fig. 2A), supporting the idea that the inhibitory effect of R54E can be suppressed by mutations that directly stimulate events at site I. We also looked in the R54E/I100T background for mutations that can activate site I × site I recombination, but do not suppress, or only partially suppress, the inhibitory effect of R54E on *resH* × *resH* recombination (i.e. white/pink colonies with the site I × site I substrate, and red/pink colonies with the *resH* × *resH* substrate; screen Z; Fig. 2A). Activating mutations that were selected as single mutations in this screen (i.e. screen ‘Z’) were all found to have low activation scores (e.g. M109I; Fig. 3, plate 6), suggesting that weak activating mutations suppress R54E less efficiently than strong mutations.

### Regulatory mutations that map to F52 and R54

**Fig. 4:**
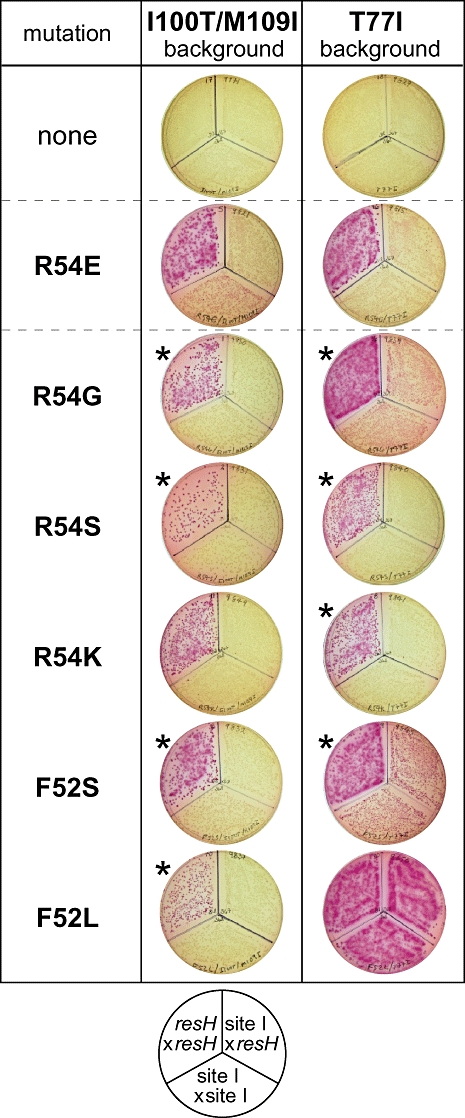
Selection of a class of Sin mutations that inhibit *resH* × *resH* recombination. Libraries of random mutants, constructed in the activated mutant backgrounds I100T/M109I and T77I (see text), were screened for mutants that can recombine a site I × site I substrate, but are defective in recombination of a *resH* × *resH* substrate. The assays show how the selected mutations, and R54E, affect recombination of *resH* × *resH*, *resH* × site I and site I × site I substrates in the I100T/M109I and T77I backgrounds; an asterisk indicates that the mutation was isolated in that background. White colonies indicate efficient recombination; red colonies indicate slow, or no, recombination.

The R54E mutation gives a distinctive regulatory phenotype (selective inhibition of *resH* × *resH* recombination) in certain activated mutant backgrounds (e.g. T77I and I100T/M109I; Fig. 3, plates 5–8). We therefore reasoned that it should be possible to select mutations in other residues involved in the same regulatory interactions. We constructed libraries with random mutations in the reading frames of T77I Sin (codons 2–66) and I100T/M109I Sin (codons 2–92, i.e. most of the core domain residues); these were screened for mutants that can recombine a site I × site I substrate, but are defective in *resH* × *resH* recombination. In total, 24 independent mutants were selected. Strikingly, all contained a mutation in one of two residues: F52 (F52L or F52S) and R54 (R54G, R54K or R54S). F52 and R54 are the two residues primarily involved in a dimer–dimer packing interface seen in the Sin crystals (Fig. 1D; Mouw *et al*., 2008). Three of the five mutations were selected in both the T77I and I100T/M109I backgrounds (Fig. 4). The mutations were all characterized in both backgrounds, and selective inhibition of *resH* × *resH* recombination was evident for every combination except F52L/T77I (Fig. 4).

In sequence alignments, Sin residues F52 and R54 correspond to γδ resolvase residues K54 and E56 which are at the regulatory 2–3′ interface (Hughes *et al*., 1990). In γδ resolvase, two further residues participate in the 2–3′ interface: R2 and R32 (Fig. 1D), which align with M1 and K29 of Sin (Fig. S1). We therefore designed mutations in these two residues. K29Q (combined with a mutation at the adjoining residue, E28S) had no significant effect on *resH* × *resH* recombination, while M1MK (lysine inserted as the second residue) virtually abolished site I × site I recombination by I100T Sin, suggesting a serious defect in catalysis (data not shown). We therefore have no evidence that residues in the N-terminal domain (NTD) other than F52 and R54 are required for activation by the regulatory module.

### Directed mutagenesis of Q115

**Fig. 5:**
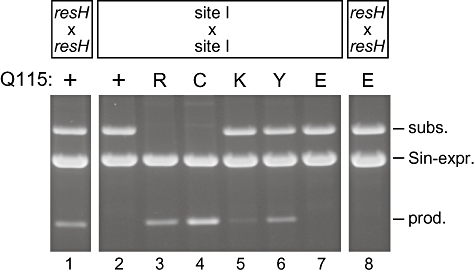
Different substitutions at residue Q115 can activate recombination. Site I × site I recombination was assayed *in vivo* with Q115 mutants (R, C, K, Y or E) and WT Sin (+); *resH* × *resH* recombination was also assayed with Q115E Sin and WT Sin. Pooled colonies from indicator plates were grown in liquid culture (∼60 generations), and uncut plasmid DNA was analysed by agarose gel electrophoresis. The substrate, product and Sin-expressing plasmids are indicated. All the mutations except Q115E activate recombination.

**Fig. 6:**
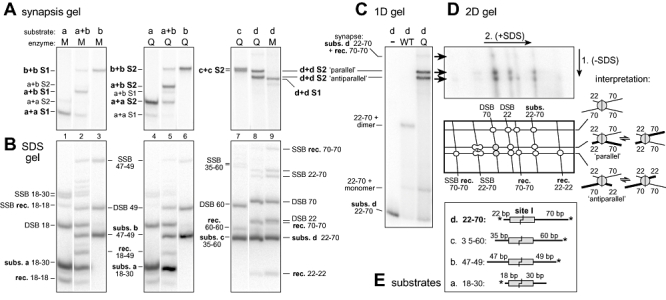
Stabilization of catalytically active site I synaptic tetramers. A. Separation of site I synaptic tetramers formed *in vitro* by the activated mutants Q115R and T77I/I100T/Q115R (Q and M respectively), by non-denaturing PAGE. The site I substrates a–d have different arm lengths, as depicted in (E). The synapse species designated S1 and S2 are named according to their component DNA substrates (e.g. b+b S2 contains two equivalents of substrate b). Reactions (24°C, 60 min) contained ∼12.5 nM site I with 125 nM Sin subunits. Only the part of the gel containing the synapses is shown (see C for a complete gel) and the lanes have been rearranged. Note that recombination intermediates present in the reaction mixtures (with mutant M in particular) may not all have been trapped as stable synapse intermediates visible on this gel (data not shown). B. Denaturing (+ SDS) gel analysis of samples from the same reactions as (A). Recombinant product and non-recombinant substrate DNA fragments (rec. and subs.) are indicated. The single-strand break (SSB) and double-strand break (DSB) products all have one Sin subunit covalently attached to the DNA, and are identified according to the attached DNA fragment. Note that when the DNA arms flanking site I are of sufficiently different lengths, the top and bottom strand SSB products migrate as a doublet of bands of equal intensity. C. Non-denaturing ‘synapsis’ gel. The Q115R (Q) reaction is similar to that in lane 8 of the gel in (A), but with ∼80 nM substrate d and 500 nM Q115R Sin. Marker reactions used ∼12 nM substrate with enzyme dilution buffer (−) or 250 nM WT Sin. Note that the synapse labelled ‘subs. d 22-70 + rec. 70-70’ is a very minor species and has the mobility expected for a synapse containing three 70 bp half-sites and one 22 bp half-site; its rarity suggests that very little dissociation/reassociation of synapses occurs during the reaction. D. Two-dimensional gel analysis of an aliquot of the sample run in (C). The first dimension is non-denaturing, and the second dimension is denaturing (+ SDS), as indicated. An interpretation of the spots, and cartoons of the presumptive synaptic complexes, are also shown. E. Structures of the site I substrates a–d. Substrates were 3′ end-labelled as shown (*); note that in the 22–70 substrate, the specific activities of the 70 and 22 ends are in the ratio ∼2:1.

Our *in vivo* data suggest that the strongest activating mutation identified is Q115R (Fig. 2A, Fig. 3). Q115 in Sin corresponds to H107 in Hin and T109 in Tn3/γδ resolvase, where activating mutations have also been identified (Fig. S1; Burke *et al*., 2004; Dhar *et al*., 2004), and it is expected to be very close to the core of the site I synaptic tetramer (Fig. 8C and D; Li *et al*., 2005). Alternative substitutions at Q115 (C, K, Y and E) were therefore made and tested *in vivo*. Q115E inhibited recombination (Fig. 5, lanes 7 and 8), although it had no significant effect on binding at site I (data not shown). Q115C, Q115K and Q115Y, like Q115R, all promoted site I × site I recombination; the order of activity is R > C > Y > K (Fig. 5, lanes 3–6). With Q115R, the resolution product has a reduced copy number (Fig. 5, lane 3); this might be due to accumulation of Q115R Sin-mediated DSBs at site I, as seen *in vitro* (Fig. 6B).

### Recombination by activated Sin mutants *in vitro*

To investigate how activating mutations alter the catalytic properties of Sin, three of the most active single mutants, T77I, K110R and Q115R, and the double mutant N72D/I100T (see Fig. 3, plates 7–14) were purified and assayed *in vitro*. These enzymes all showed a topological selectivity similar to that of WT Sin in reactions with a supercoiled *resF*^AT^ × *resF*^AT^ substrate and IHF as the cofactor, giving primarily two-node catenane resolution products (Fig. S2; Fig. 7, lane 13). (The *resF*^AT^ substrate has AT as the central dinucleotide at site I, allowing the possibility of both resolution and inversion reactions; Rowland *et al*., 2006.) Similar results were obtained using a *resH*^AT^ substrate and Hbsu (*Bacillus subtilis* HU) as the cofactor (data not shown). Since the two-node catenane resolution product is diagnostic for the −3 synaptosome (Fig. 1B), the data indicate that the activating mutations do not prevent normal synaptosome assembly (Rowland *et al*., 2002; 2005).

Unlike WT Sin, the activated mutants all recombined a site I^AT^ × site I^AT^ substrate (i.e. in the absence of site II and Hbsu/IHF), by the unregulated ‘random collision’ pathway previously characterized with I100T Sin (Rowland *et al*., 2005). Resolution and inversion products were formed in equal yield (Fig. 7, lane 1; Fig. S2), presumably derived from synapses with sites in ‘parallel’ and ‘antiparallel’ alignments. Topological analysis of the products revealed a mixture of simple and complex supercoiled catenanes and knots (Fig. S2), as seen previously with I100T Sin (Rowland *et al*., 2005), indicating that strand exchange takes place within a site I synapse.

### Activating mutations stabilize catalytically active site I^AT^ synapse intermediates

Activating mutations in Sin abolished the requirement for (−) supercoiling, allowing recombination intermediates to be observed directly using short linear site I^AT^ substrates (Fig. 6). We present data for two Sin mutants, Q115R and T77I/I100T/Q115R, which combines three activating mutations (abbreviated Q and M in Fig. 6). Both mutants formed site I^AT^ synapses that could be trapped and separated by non-denaturing PAGE (Fig. 6A), and that migrate much more slowly than the site I^AT^–dimer complex seen with WT Sin (Fig. 6C). With the triple mutant, a single major species, designated S1, was seen with any given substrate (Fig. 6A, lanes 1 and 3, and data not shown; see Fig. 6E for substrate structures). In contrast, with Q115R Sin, a distinct slower-migrating species, S2, was seen, although species that co-migrate with the S1 synapse were also detected (Fig. 6A, lanes 4 and 6). When two substrates of different lengths were mixed, additional species of intermediate mobility appeared (Fig. 6A, lanes 2 and 5), confirming that S1 and S2 are synapses containing two copies of site I^AT^. When the left and right arms of the site I^AT^ substrate were of very different lengths (i.e. substrates c and d; Fig. 6E), the Q115R S2 synapse ‘split’ into a doublet of bands of equal intensity (Fig. 6A, lanes 7 and 8). We interpret these as the ‘parallel’ and ‘antiparallel’ isomers (see Fig. 6D), which presumably separate on gels only when the DNA arm lengths are sufficiently different. All of these site I^AT^ synapses are thought to contain a tetramer of Sin, because their gel mobility properties resemble those of Tn3/γδ resolvase site I synapses (data not shown), for which the stoichiometry is known (Sarkis *et al*., 2001; Nöllmann *et al*., 2004; Olorunniji *et al*., 2008), and because Q115R Sin, unlike WT Sin, can form a stable apo-tetramer and a site I synaptic tetramer in solution (K.W. Mouw *et al*., submitted).

To follow the progress of site I^AT^ × site I^AT^ recombination in the synapsis reactions analysed on the non-denaturing gel (Fig. 6A), samples were also quenched with SDS and analysed by SDS-PAGE (Fig. 6B). Both Q115R Sin and T77I/I100T/Q115R Sin generated recombinants of the predicted sizes, together with presumptive recombination intermediates in which Sin subunits were covalently linked to the DNA fragments. DSB intermediates accumulated to higher levels than single-strand break (SSB) intermediates, which represent partially cut substrates and partially re-ligated recombinants (Fig. 6B). The identities of the various species were confirmed by observing the effects of altering the arm lengths of the substrate (as in Fig. 6B), and digesting with proteinase K (data not shown).

To investigate the composition of individual Q115R Sin synapse species isolated by non-denaturing PAGE, a reaction similar to that in lane 8 of Fig. 6A was analysed by 2D-PAGE (Fig. 6C and D). This showed that the ‘parallel’ and ‘antiparallel’ synapses consisted mainly of DSB intermediates, with Sin subunits covalently joined to the DNA (Fig. 6D, DSB 70 and DSB 22). Both synapses also contained uncut substrate (22–70), and smaller amounts of SSB intermediates (SSB 22–70). Only the faster-migrating synapse, thought to represent the ‘antiparallel’ isomer, contained detectable amounts of the fully ligated recombinants (22–22 and 70–70) and an incompletely ligated recombinant (SSB 70–70). Note that the ‘parallel’ synapse is not expected to contain detectable recombinants (since they will be the same sizes as the substrates). A simple interpretation of these data is that each Q115R synapse ‘species’ separated on the gel is in fact an equilibrating ensemble of all the catalytic intermediates, starting from a specific alignment of the uncut substrates, and proceeding through the SSB and DSB intermediates to the corresponding synapsed recombinants (see Fig. 6D). Consistent with this scenario, the ‘antiparallel’ synapse contains roughly comparable amounts of uncut substrate and ligated recombinant; however, other explanations cannot be ruled out.

The fast-migrating synapses designated S1 in Fig. 6A (seen with T77I/I100T/Q115R Sin, and to a lesser extent with Q115R Sin) are candidates for an ‘early’ or pre-cleavage synapse intermediate. In support of this, synapses co-migrating with these S1 synapses were also formed in the absence of detectable DNA strand cleavage, for example, with the activated mutant T77I Sin (data not shown); the recombination intermediates detected with T77I/I100T/Q115R Sin in Fig. 6B are thought to derive not from the S1 synapses seen in Fig. 6A, but from unstable synapses that dissociate during electrophoresis (data not shown). Interestingly, the S1 synapse did not ‘split’ into a doublet with the 22–70 substrate (substrate d; Fig. 6A, lane 9), in contrast to the S2 synapse (Fig. 6A, lane 8). This could be due to a different configuration of the DNA arms in the synapse; alternatively, the parallel and antiparallel isomers might interconvert rapidly and thus migrate as a single species.

### The mutation R54E interferes with the function of the regulatory module

To investigate the role of the F52/R54 interface in regulatory site function, we examined the effects of the mutation R54E on *in vitro* recombination by an activated Sin mutant, N72D/I100T (Fig. 7). This mutant was chosen because it catalyses site I^AT^ × site I^AT^ recombination *in vitro* at an easily measurable rate (see Fig. 3, plates 9 and 10 for *in vivo* data). Both N72D/I100T Sin and R54E/N72D/I100T Sin recombined a site I^AT^ × site I^AT^ substrate relatively slowly (Fig. 7, lanes 1–4), giving approximately equal amounts of resolution and inversion products and some intermolecular product, characteristic of an unregulated ‘random collision’ reaction (Rowland *et al*., 2002). Although the R54E mutation had no significant qualitative effect on recombination, it reduced the rate slightly (consistent with its effect on I100T Sin *in vivo*; Fig. 3, plates 3 and 4). (IHF was present in these reactions, but does not affect the products; data not shown.)

When site II was present (i.e. with a *resF*^AT^ × *resF*^AT^ substrate), but IHF was absent, recombination by both N72D/I100T Sin and R54E/N72D/I100T Sin was slower than with the site I^AT^ × site I^AT^ substrate (Fig. 7, lanes 5–8). This suggests that synapsis at site II inhibits recombination at site I when there is no IHF to bend the site I–site II spacer (see Fig. 9A and *Discussion*). When a low concentration of IHF was added, N72D/I100T Sin and R54E/N72D/I100T Sin responded very differently (Fig. 7, lanes 9–12). With N72D/I100T Sin, 0.5 μg ml^−1^ IHF strongly stimulated resolution, but not inversion, at initial times [Fig. 7, compare lanes 1 (no site II), 5 (no IHF) and 9 (site II + IHF)]. (Inversion product accumulated at later times, and may be a secondary reaction product.) In contrast, with R54E/N72D/I100T Sin, 0.5 μg ml^−1^ IHF did not significantly stimulate resolution; the recombination products were similar to those seen with the site I^AT^ × site I^AT^ substrate (Fig. 7, compare lanes 3–4 and 11–12), and raising the enzyme concentration had no effect (data not shown). The R54E mutation therefore largely blocked stimulation by 0.5 μg ml^−1^ IHF. However, when the concentration of IHF was raised to 3.0 μg ml^−1^, the effect of R54E was overcome, and resolution was selectively stimulated (Fig. 7, compare lanes 11–12 and 15–16). Similar results were obtained with a *resF*^AC^ × *resF*^AC^ substrate, when only resolution is possible: R54E abolished recombination at 0.5 μg ml^−1^ IHF (Fig. 7, compare lanes 17–18 and 19–20), but inhibited only weakly at 3.0 μg ml^−1^ IHF (Fig. 7, compare lanes 21–22 and 23–24).

In summary, at a low concentration of IHF, the R54E mutation has a dramatic inhibitory effect on the resolution reaction, whereas at a high concentration of IHF, it has little effect. Similar results were obtained with a *resH*^AT^ × *resH*^AT^ substrate and Hbsu as the cofactor (data not shown). The main effect of R54E is thus to raise the apparent ‘K_M_’ for the DNA-bending cofactor in the resolution reaction (i.e. the concentration required for half-maximal stimulation).

## Discussion

### Regulatory mutations in Sin

The objective of the random mutagenesis studies reported here was to identify protein interfaces that are involved in regulating recombination by Sin, thus providing insight into the architecture and function of the synaptosome. Our structure-based model of the synaptosome (Mouw *et al*., 2008) calls for three distinct types of Sin dimer–dimer interactions: (A) to assemble a ‘DNA-out’ synaptic tetramer at site I, (B) to mediate contact between the dimers bound at site I and site II, and (C) to assemble a ‘DNA-in’ synaptic tetramer at site II (Figs 1C and 9E and F). We have now identified regulatory mutations that influence each of these three proposed interactions, and that map to specific crystallographic interfaces, providing strong experimental support for the model. Previously, we described a class of mutations that inhibit recombination by inhibiting site II synapsis, and that map to the crystallographic CTD interface at site II (Mouw *et al*., 2008). Here, we describe two other classes of Sin regulatory mutations, affecting interactions of types (A) and (B) respectively: activating mutations, which bypass the requirement for the regulatory sites and stimulate recombination at site I, and mutations at F52 and R54, which reverse the activating effect of the regulatory sites on recombination at site I. The F52 and R54 mutations (Fig. 4) map to a dimer–dimer packing interface seen in the Sin crystals (Fig. 1D; Mouw *et al*., 2008). These data, and the *in vivo* and *in vitro* properties of R54E mutants (Figs 3 and 7), strongly support our proposal that the F52/R54 interface stimulates recombination at site I by mediating direct contacts between the regulatory and catalytic subunits (Mouw *et al*., 2008). As we discuss below, the activating mutations highlight residues likely to be involved in the large-scale conformational rearrangements needed to assemble the active catalytic tetramer at site I.

### Activating mutations

Activating mutations are defined here as those that stimulate site I × site I recombination, bypassing the normal requirement for the regulatory sites. A total of 36 activating mutations in Sin were identified; overlapping sets of these were selected in screens based on *resH* × *resH* recombination, on site I × site I recombination, or on the suppression of R54E-mediated inhibition of *resH* × *resH* recombination (screens W, X, Y and Z; Fig. 2A). To account for this, we suggest that all activating mutations directly stimulate events at site I that normally depend on the regulatory sites and the F52/R54 interface, and that the activating mutations and the regulatory sites can work together to promote recombination. Our data indicate that any Sin mutation that stimulates site I × site I recombination (i.e. in the absence of the regulatory sites) will also stimulate *resH* × *resH* recombination within the synaptosome, and vice versa; the one exception found is the CTD mutation H166R, which does not stimulates site I × site I recombination, and is thought to act by stabilizing the site II synapse (Mouw *et al*., 2008).

### Activating mutations and synapsis at site I

Activating mutations were found at 27 of the 146 residues in the N-terminal catalytic domain of Sin (Fig. 2A). Of these, Q115R had the strongest effects, giving a high level of site I × site I recombination (Figs 3 and 5, Fig. S2), and stabilizing an active site I synaptic tetramer (Fig. 6) and a solution apo-tetramer (K.W. Mouw *et al*., submitted) (neither tetramer has been detected with WT Sin). For Tn3 and γδ resolvases, in contrast, combinations of three or more activating mutations (Arnold *et al*., 1999; Burke *et al*., 2004) were needed to trap a synaptic tetramer (Sarkis *et al*., 2001; Nöllmann *et al*., 2004; Li *et al*., 2005), or apo-tetramer (Kamtekar *et al*., 2006), for structural and biochemical studies. In the Sin system, combinations of activating mutations have additive effects on site I × site I recombination in all cases tested (e.g. see Fig. 3, plates 3 and 5; data not shown), suggesting that these mutations all contribute to the same process. The positions of activating mutations in Sin correlate remarkably well with those in Tn3 and γδ resolvases, and with mutations conferring FIS independence in the DNA invertases Hin and Gin (Fig. S1; Klippel *et al.*, 1988; Haykinson *et al*., 1996; Johnson, 2002; Burke *et al*., 2004). This supports the idea that in all these systems, the regulatory sites control recombination by stabilizing similar catalytic intermediates, and helps to justify our use of the activated γδ resolvase tetramer structures (Li *et al*., 2005; Kamtekar *et al*., 2006) in modelling the site I component of the Sin synaptosome (Figs 1C, 8C and 9E and F; Mouw *et al*., 2008).

To picture the likely effects of activating mutations in the Sin site I synaptic tetramer, we examined the corresponding positions in the γδ resolvase tetramer structures, and we also modelled a Sin tetramer by fitting segments of the Sin site II dimer structure onto the γδ resolvase structure (Fig. 8C). The Sin site II dimer structure (Fig. 8A) (Mouw *et al*., 2008) is thought to be a good model for the pre-synapsis site I dimer within the main part of the catalytic domain (residues 1–126), where the two Sin subunits adopt conformations very similar to those seen in γδ resolvase dimers. When mapped onto the Sin site II dimer structure, the activating mutations do not define a single surface patch or a plausible synapsis interface (Fig. 8A). In particular, they do not correlate with a pseudo-symmetric ‘DNA-out’ dimer–dimer packing interface seen in the Sin site II crystals (Mouw *et al*., 2008), which closely resembles the ‘DNA-out’ synapsis interface proposed in models of the Tn3/γδ resolvase synaptosome (Sarkis *et al*., 2001; Nöllmann *et al*., 2004). While we cannot rule out the possibility that Sin site I synapsis is initiated by dimer–dimer contacts of this type, analogous to those modelled *in silico* for γδ resolvase (Li *et al*., 2005), our data provide no support for the idea.

To explain how mutations at ∼20% of Sin NTD residues can activate recombination at site I, substituting for the function of the regulatory module, we suggest a simple ‘two-state’ model for synapsis at site I; the supporting evidence is described below. We hypothesize that a Sin dimer bound at site I can interconvert between two major conformational states: a ‘closed’ conformation similar to that seen in the γδ resolvase pre-synapsis site I dimer (Yang and Steitz, 1995) and in the Sin site II dimer (Fig. 8A; Mouw *et al*., 2008), and an ‘open’ conformation (which may be short-lived) similar to that seen in the γδ resolvase synaptic tetramer (Fig. 8B; Li *et al*., 2005; Kamtekar *et al*., 2006). We propose that the ‘closed’ dimer is refractory to synapsis, whereas the ‘open’ dimer exposes a high-affinity synapsis interface needed to assemble the synaptic tetramer (Fig. 8B and C). According to this model, activating mutations could promote synapsis by destabilizing the ‘closed’ dimer, and/or by stabilizing the ‘open’ dimer or the synaptic tetramer itself.

The above model is supported by the following observations. Most of the activating mutations in Sin affect residues on or close to internal domain interfaces that are expected to stabilize the ‘closed’ dimer, but need to be broken or rearranged to form the ‘open’ dimer (Fig. 2A; Fig. 8A and B). For example, various substitutions at Q115, which is at the dimer interface, all activated Sin (R, C, K and Y; Fig. 5). A number of activating mutations (including three of the strongest, Q115R, K110R and V78A; Fig. 2A) map to interfaces within the ‘closed’ dimer that are rearranged to create the dimer–dimer synapsis interface in our model of the Sin tetramer (mainly one face each of the D, D′ and E helices; Fig. 8). The Q115R mutation could stabilize the tetramer by placing a basic residue close to an acidic residue (E122) across the synapsis interface (Fig. 8D). In contrast, the Q115E mutation was predicted to generate an unfavourable configuration of negatively charged residues across the synapsis interface, and was found to abolish recombination (Fig. 5). Also notable is the cluster of activating mutations on one face of helix D (residues 72, 75, 78, 82 and 83). In the ‘closed’ dimer, these residues are involved in the ‘cis’ interface between the core catalytic domain and helix E of the same subunit (Fig. 8A); in the hypothesized ‘open’ dimer, the interface is rearranged (Fig. 8B) such that residues N72 and I75 can now contribute to the synapsis interface in the tetramer (Fig. 8D), as seen for the corresponding residues (A74 and I77) in γδ resolvase (Li *et al*., 2005). Analysed in this way, the locations of these and other activating mutations appear consistent with the idea that their effect is to destabilize the pre-synaptic dimer relative to the synaptic tetramer. In the Tn3 system, an intra-subunit disulphide cross-link that should favour an ‘open’ dimer conformation is strongly activating (Wenwieser, 2001; Kamtekar *et al*., 2006), supporting our proposal that a conformational change in the site I-bound subunits is sufficient to trigger synapsis.

### Activating mutations stabilize a catalytically proficient site I synapse

The activating mutation Q115R stabilizes a site I synapse with the properties of an authentic recombination intermediate. First, ‘parallel’ and ‘antiparallel’ isomers, formed in approximately equal yield, could be separated by PAGE (Fig. 6A), consistent with previous evidence that crossover site alignment is random in the absence of the regulatory sites (Rowland *et al*., 2005). Second, although each synapse isomer migrated as a well-defined band in PAGE, it contained site I in different states of DNA strand cleavage (Fig. 6D), suggesting that the strand cleavage and re-ligation steps may equilibrate within the synapse. Third, in the ‘antiparallel’ synapse, comparable amounts of recombinant and non-recombinant site I were detected (Fig. 6D), suggesting that the strand exchange steps also equilibrate on the timescale of the PAGE separation (we would otherwise expect to separate ‘recombinant’ and ‘non-recombinant’ isomers of the synapse). We therefore conclude that all the chemical and conformational steps of recombination take place within the Q115R Sin synaptic tetramer, and may be in a dynamic equilibrium. This contrasts with previous reports of synaptic complexes trapped with activated mutants of γδ resolvase or Hin invertase, where DNA strand cleavage was quantitative (Dhar *et al*., 2004; Sanders and Johnson, 2004; Li *et al*., 2005; Kamtekar *et al*., 2006).

Activating mutations in Sin other than Q115R (e.g. T77I, I100T and K110R) also promoted site I synapsis (Fig. 6A; data not shown), and topological studies using supercoiled substrates confirmed that site I × site I recombination takes place within a site I synapse (Fig. S2), as demonstrated previously for I100T Sin (Rowland *et al*., 2005). Although our data support the idea that all activating mutations promote synapsis at site I, they may not all stabilize an ‘S2’ synapse intermediate similar to that characterized with Q115R Sin. Specifically, T77I-containing mutants stabilized an ‘S1’ synapse, which appears to have a different conformation (Fig. 6A), and may be an earlier (pre-cleavage) intermediate. We anticipate that the various activated mutants will be useful for trapping different post-synapsis intermediates for biochemical and structural studies.

### Role of the F52/R54 interface

In the Sin site II crystal structure, each Sin dimer contacts two neighbours using a pseudo-symmetric interface centred on residues F52 and R54 (Mouw *et al*., 2008). Our data argue that this F52/R54 interface has an important role in promoting recombination at site I within the synaptosome. Previously, we showed that a designed substitution in Sin, R54E, can selectively block regulatory site functions (Mouw *et al*., 2008). We have now selected a class of regulatory mutations with similar properties to R54E and shown that they map exclusively to the two major residues in the crystallographic F52/R54 dimer–dimer interface (Fig. 4). Mutations were not found in a third residue, D57, which contacts R54 across the interface (Fig. 1D); this residue is very highly conserved (Fig. S1) and may be essential for the secondary structure.

Our initial structure-based model of the Sin synaptosome, assembled by rigid-body docking of the available crystal structures, is not compatible with formation of the F52/R54 interface between the Sin dimers bound at sites I and II (Figs 1C and 9F; Mouw *et al*., 2008). However, the F52/R54 interface can be incorporated into the model by making conformational adjustments that include a rotation of the Sin catalytic domains at site II towards the catalytic tetramer (compare Fig. 9E and F; Mouw *et al*., 2008). We suggest that the synaptosome adopts a conformation of this type during at least a part of the catalytic cycle.

### Mechanism of regulation

We argued above that the regulatory module is likely to stimulate the same critical reaction steps at site I as the activating mutations. We have shown that activating mutations can promote site I synapsis, and that the resulting site I synapse is proficient in recombination, at least in the case of Q115R Sin (Fig. 6D). Recent studies using a novel suicide substrate have demonstrated that a change in the multimeric state of Sin is an essential step in switching on catalysis (K.W. Mouw *et al*., submitted). We have also shown that F52 and R54 are important for the control of recombination by the regulatory module (Figs 4 and 7). Our model of the synaptosome suggests a specific mechanism whereby F52/R54 contacts (Fig. 1D) could promote site I synapsis, and hence recombination, by mediating a ‘looping’ interaction between Sin dimers bound at sites I and II within each *resH*/*resF* site (Fig. 9). We propose that this interaction stabilizes the ‘open’ conformation of the site I dimer, which we suggest is required for assembly of the site I tetramer; modelling suggests that looping would not be compatible with a ‘closed’ dimer at site I. The looping interaction is expected to require both the F52/R54 interface and the HU/IHF-induced DNA bend (Fig. 9C and D). We would therefore predict that, in the absence of HU/IHF, site II synapsis would simply ‘tether’ the site Is in an unfavourable configuration for synapsis (Fig. 9A), and that any defect in the F52/R54 interface would have a more severe effect on recombination at low concentrations of HU/IHF. This prediction is borne out by our *in vitro* data, which show that at a low concentration of HU/IHF (but not at a high concentration) a WT F52/R54 interface is essential for the stimulatory effect of the regulatory sites on recombination (Fig. 7). The *in vitro* behaviour at low HU/IHF may reflect the situation *in vivo*, where the selective inhibition of *resH* × *resH* recombination by R54E is particularly clear (Fig. 3).

In summary, in our working model for regulation, the ‘closed’ dimer conformation of WT Sin bound at site I is resistant to synapsis and recombination. Synapsis at site II also helps to repress recombination, except when the regulatory module is active, i.e. when the F52/R54 interface and HU/IHF cooperate to ‘open’ the site I dimers and assemble the catalytic tetramer (Fig. 9A). This type of model for allosteric activation nicely fits the available genetic, biochemical and structural data, in particular our evidence that the activating mutations and the F52/R54 interface can work together to stimulate recombination. However, we cannot exclude the possibility that the regulatory module and the F52/R54 interface act simply to ‘recruit’ a high local concentration of correctly aligned site I-bound dimers, and play no further part in assembly of the catalytic tetramer.

Our model for regulation may be relevant to other serine recombinases, particularly the major families of resolvases, in which residues corresponding to the F52/R54 interface of Sin and the 2–3′ interface of Tn3/γδ resolvase are well conserved (Fig. S1; Mouw *et al*., 2008). Since these interfaces have very similar geometries (Fig. 1D), we suggest that looping interactions within *res* sites (e.g. as described for γδ*res*; Salvo and Grindley, 1988) could bring the regulatory subunits into direct contact with the catalytic subunits, in positions similar to those shown in our model for Sin (Fig. 9E).

## Experimental procedures

### Mutagenesis of Sin and selection of activated mutants

The construction of PCR-generated libraries of random mutants (Zaccolo *et al*., 1996) of WT Sin has been described (libraries 7.1 and 7.4, both ∼600 000; Mouw *et al*., 2008). Libraries of mutants of I100T Sin (∼300 000) and R54E/I100T Sin (∼600 000) were constructed in a similar way. The substrates used to assay recombination *in vivo* (*resH* × *resH*, *resH* × site I and site I × site I), the complementation assay (see text for a summary), and the method used to estimate percentage resolution, have been described (Rowland *et al*., 2005; Mouw *et al*., 2008).

### Selection of mutations that selectively inhibit resH × resH recombination

Libraries with mutations in codons 2–92 of the T77I reading frame, or codons 2–66 of the I100T/M109I reading frame, were constructed by replacing appropriate restriction fragments with corresponding randomly mutagenized restriction fragments from a 1:1 mixture of the two WT Sin library DNAs (see above). From the new libraries, sub-libraries (size 1400−1650) were made by selecting mutants that are defective in recombination of pSB(*resH* × *resH*) (i.e. that give red or pink colonies in the *in vivo* assay); the sub-libraries were then screened for mutants that can recombine pSB(site I × site I) (i.e. white colonies in the *in vivo* assay).

### *In vitro* recombination assays

His-tagged WT Sin and mutant derivatives were overexpressed in *E. coli* and purified as described previously (Rowland *et al*., 2005), or on a small scale as follows. Cells (∼0.1 g) were resuspended in 1 ml of buffer P (25 mM sodium phosphate pH 7.8, 10 mM β-mercaptoethanol, 0.1 mM EDTA, 1.2 mM PMSF) supplemented with 100 mM NaCl and 1 mM EDTA, and broken by freeze–thaw cycles in liquid nitrogen. After centrifugation, the insoluble material was successively extracted with buffer P supplemented with (i) 200 mM NaCl, (ii) 400 mM NaCl, (iii) 2 M NaCl, (iv) 1% Triton X-100 and (v) 6 M urea. The urea fraction was bound to SP sepharose, and Sin was eluted with a step of 0.4 M NaCl, then bound to Ni-NTA agarose (Qiagen) in the presence of 5 mM imidazole, 5 M urea and 0.7 M NaCl. After renaturation *in situ* with a step of 2 M NaCl, 25 mM sodium phosphate pH 7.8, 20 mM imidazole, Sin was eluted in the same buffer at 250 mM imidazole. Fractions were stored at −20°C after adding glycerol to 50% (v/v).

Sin concentrations were estimated from SDS-PAGE, using a reference sample of γδ resolvase of known concentration (from absorbance at 280 nm). DNA concentrations were estimated from the absorbance at 260 nm.

The site I^AT^, *resF*^AT^ and *resF*^AC^ substrates were described previously (Rowland *et al*., 2005; 2006). For recombination assays with supercoiled DNA, reaction mixtures contained 50 mM Tris/HCl pH 8.2, 100 mM NaCl, 10 mM MgCl_2_, 5% glycerol, 4% Ficoll, 0.1 mM EDTA, 20–24 μg ml^−1^ (8–10 nM) plasmid DNA substrate, with 250–300 nM Sin subunits and 0.5–3.0 μg ml^−1^ IHF (23–140 nM) or HU (Hbsu). Reactions were typically initiated by adding 2 μl of Sin to 20 μl of assay mixture at ∼22°C, and were terminated by heating (80°C, 5 min). Samples were nicked with DNase I, or cut with XhoI, and were treated with SDS and proteinase K prior to agarose gel electrophoresis.

### PAGE analysis of site I synaptic tetramers

For recombination/synapsis assays with short linear DNA substrates, reaction mixtures contained 20 mM Tris/HCl pH 7.5, 100 mM NaCl, 12.5 mM imidazole, 9% glycerol, 4% Ficoll, 10 μg ml^−1^ poly [dI/dC] (Sigma), 10–20 nM site I DNA substrate, with 100–250 nM Sin subunits. The substrates were ^32^P-end-labelled restriction fragments (45–100 bp) purified by PAGE. (Specific activities of the two ends may differ; for the ‘35–60’ substrate, only one end was labelled.) Reactions were typically initiated by adding 2 μl of Sin to 20 μl of assay mixture at ∼22°C, and after 5–60 min were placed on ice before loading 6 μl of samples onto non-denaturing MOPS gels [5% polyacrylamide (29:1 acrylamide:(bis)acrylamide), 100 mM MOPS/NaOH pH 7.0, 0.1 mM EDTA]. Gels were pre-run for 30 min (∼10–15 V cm^−1^, 4°C), and run for ∼3 h without tracking dyes or buffer recirculation, using gel kits with 500 ml buffer reservoirs. For SDS-PAGE, reactions were quenched by adding SDS (0.1% final), and 3–4 μl of samples were loaded onto TBE polyacrylamide gels [6% polyacrylamide (29:1 acrylamide:(bis)acrylamide), 0.1% SDS, 100 mM Tris, 100 mM borate, 0.1 mM EDTA/NaOH; pH ∼8.3]. Gels were dried and phosphorimaged by standard methods.

For the 2D-PAGE experiment (Fig. 6D), higher concentrations of the ‘22–70’ site I substrate (80 nM) and Q115R Sin (500 nM) were used. After 60 min, a 6 μl sample of the reaction was loaded near one edge of a 5% non-denaturing MOPS gel and electrophoresed as above; the side-spacers were then removed, the gel was turned through 90° without removing the glass plates, and electrophoresis was continued (initially at 4°C) in the same buffer plus 0.1% SDS, for 105 min at 10 V cm^−1^. Note that because the gel is not soaked in SDS after the first dimension, the first few minutes of the second dimension electrophoresis are under non-denaturing conditions; DNA fragments or covalently linked protein–DNA complexes released from their synapses by SDS are separated in the second dimension from the ‘smear’ of DNA fragments released by dissociation of protein–DNA complexes during the first dimension.

### Molecular modelling

All of the molecular models, and the figures, were made using ‘Pymol’ (DeLano, 2002).
