# Supplementary material for: Regulatory mutations in Sin recombinase support a structure-based model of the synaptosome
Source: Mol Microbiol. 2009 Jun 23;74(2):282–98. doi: 10.1111/j.1365-2958.2009.06756.x (PMC2764113; doi:10.1111/j.1365-2958.2009.06756.x)
Supplement: Supplementary file 1 [file mmi0074-0282-SD1.pdf]

SUPPLEMENTARY FIGURES AND LEGENDS

Figure S1

The positions of activating mutations in Sin and related resolvases and DNA invertases are closely correlated.

A structure-based alignment of the Sin, Tn3 resolvase, [illegible] resolvase, Hin and Gin sequences, highlighting residues (green) where activating mutations have been reported from random mutagenesis screens (pale green: positions where activating mutations were effective in a specific multiple mutant) (Table 1A; Klippel *et al.*, 1988; Haykinson *et al.*, 1996; Burke *et al.*, 2004; Johnson, 2002). Secondary structure elements for Sin (Mouw *et al.*, 2008) and [illegible] resolvase (Yang and Steitz, 1995) are shown, and residues conserved in all of the enzymes shown are in grey. Also highlighted are residues involved in the F52/R54 and 2-3' interfaces of Sin and [illegible] resolvase (orange), and residues implicated in the CTD synapsis interface of Sin (cyan, except for H166 [magenta]) (Mouw *et al.*, 2008). Sin residues R54 and Q115 are marked, as are Sin and [illegible] resolvase residues referred to in the legend to Fig. 7C (to describe the segments used to construct the Sin site I tetramer model).

Figure S2

Topological analysis of Sin *in vitro* reaction products.

A Recombination of a supercoiled site I^{AT} x site I^{AT} substrate by a panel of activated Sin mutants, showing recombinant products with complex knotted and catenated topologies. Substrate supercoils trapped between the sites have therefore been converted into knot/catenane nodes in the recombinants, providing clear evidence that the four DSB ends are formed and re-ligated within a site I synapse. (If the DSB ends were not confined within a synapse, substrate supercoiling would be relaxed prior to re-ligation of the recombinants.) Reactions were nicked with DNase I and run on a 0.7% agarose gel to analyse the topological complexity of the products. Species assigned as unknotted circular substrate (0), and knot and catenane products with up to five nodes are clearly visible; more complex products are also seen. Unlinked circular resolution products are marked, as are linear DNAs of the same size (lin.), which are the expected products if Sin makes DSBs at both copies of site I. Reactions (22° C, 80 min) contained 24 [illegible]g/ml supercoiled plasmid substrate and the following Sin derivatives at ~250 nM (unless stated): (1) no enzyme; (2) WT; (3) I100T; (4) T77I; (5) K110R (375 nM); (6) Q115R; (7) N72D/I100T; (8) R54E/N72D/I100T (300 nM); (9) T77I/I100T/Q115R.

Products with 2, 4 or 6 nodes are likely to be catenated resolution products, while products with 3, 5 or 7 nodes are likely to be knotted inversion products. Restriction analysis (not shown) confirmed that resolution and inversion products were present in amounts consistent with this interpretation, and analysis of uncut samples (not shown) confirmed that many of the recombinants were topologically closed and retained negative supercoiling.

B Recombination of a supercoiled *resF*^{AT} x *resF*^{AT} substrate by the same panel of activated Sin mutants, in the presence or absence of IHF (3 μg/ml). Reactions were nicked with DNase I to reveal the 2-noded catenane resolution product characteristic of reactions involving the complete synaptosome (the reactions are slightly over-nicked, therefore unlinked circular resolution products are overrepresented). Catenanes and knots are labelled as in **A**. Reactions were as above, but for 60 min with 20 μg/ml supercoiled plasmid substrate. The enzymes used are as in **A** (1-8). Note that at the high concentration of IHF used here, the R54E/N72D/I100T enzyme gave mainly 2-noded catenane resolution products, consistent with the result shown in Fig. 6, lane 16. Restriction analysis (not shown) confirmed that with all the activated Sin mutants, resolution was specifically stimulated by IHF; in reactions without IHF, resolution and inversion were equally efficient.

Fig. S1

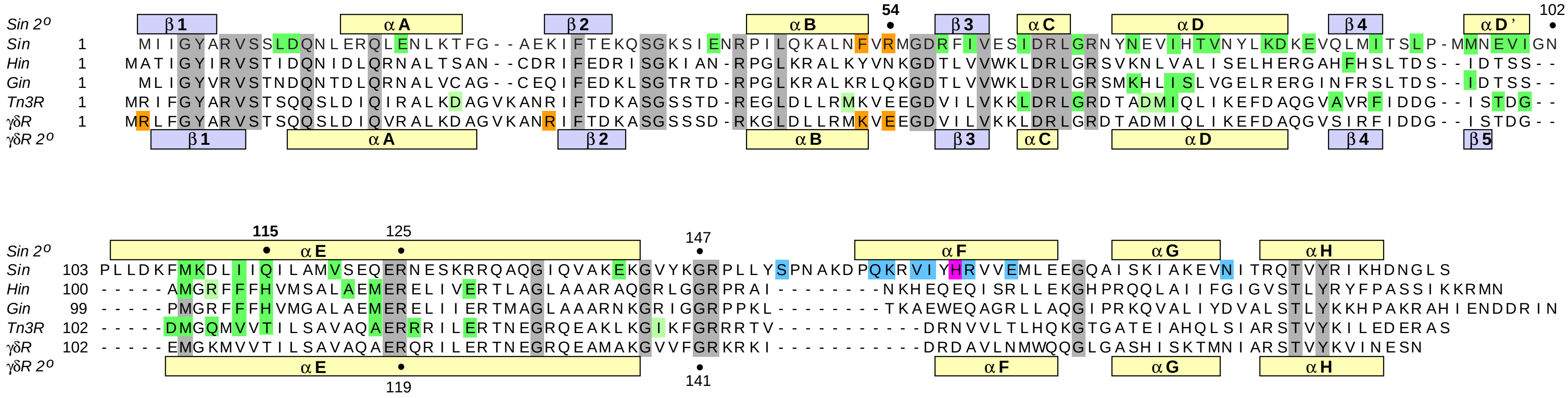

Sin 2º
β1 αA β2 αB 54 β3 αC αD β4 αD' 102
Sin 1 MIIGYARVSSLDQNLERQLENLKTFG--AEKIFTEKQSGKSIENRPILQKALNFVRMGDRFIVESIDRLGRNYNEVIHTVNYLKDKEVQLMITSLP-MMNEVIGN
Hin 1 MATIGYIRVSTIDQNIDLQRNALTSAN--CDRIFEDRISGKIAN-RPGLKRALKYVNKGDTLVVWKLDRLGRSVKNLVALISELHERGAHFHSLTDS--IDTSS--
Gin 1 MLIGYVRVSTNDQNTDLQRNALVCAG--CEQIFEDKLSGTRTD-RPGLKRALKRLQKGDTLVVWKLDRLGRSMKHLISLVGELRERGINFRSLTDS--IDTSS--
Tn3R 1 MRIFGYARVSTSQQSLDIQIRALKDAGVKANRIFTDKASGSSTD-REGLDLLRMKVEEGDVILVKKLDRLGRDTADMIQLIKEFDAQGVAVRFIDDG--ISTDG--
γδR 1 MRLFGYARVSTSQQSLDIQVRALKDAGVKANRIFTDKASGSSSD-RKGLDLLRMKVEEGDVILVKKLDRLGRDTADMIQLIKEFDAQGVSIRFIDDG--ISTDG--
γδR 2º
β1 αA β2 αB β3 αC αD β4 β5
Sin 2º
115 125 147
αE αF αG αH
Sin 103 PLLDKFMKDLIIQILAMVSEQERNESKRRQAQGIQVAKEKGVYKGRPLLYSPNAKDPQKRVIYHRVVEMLEEGQAISKIAKEVNITRQTVYRIKHDNGLS
Hin 100 -----AMGRFFFHVMSALAEMERELIVERTLAGLAAARAQGRLGGRPRAI---------NKHEQEQISRLLEKGHPRQQLAIIFGIGVSTLYRYFPASSIKKRMN
Gin 99 -----PMGRFFFHVMGALAEMERELIIERTMAGLAAARNKGRIGGRPPKL---------TKAEWEQAGRLLAQGIPRKQVALIYDVALSTLYKKHPAKRAHIENDDRIN
Tn3R 102 -----DMGQMVVTILSAVAQAERRRILERTNEGRQEAKLKGIKFGRRRTV------------DRNVVLTLHQKGTGATEIAHQLSIARSTVYKILEDERAS
γδR 102 -----EMGKMVVTILSAVAQAERQRILERTNEGRQEAMAKGVVFGRKRKI------------DRDAVLNMWQQGLGASHISKTMNIARSTVYKVINESN
γδR 2º
αE αF αG αH
119 141

Fig. S2

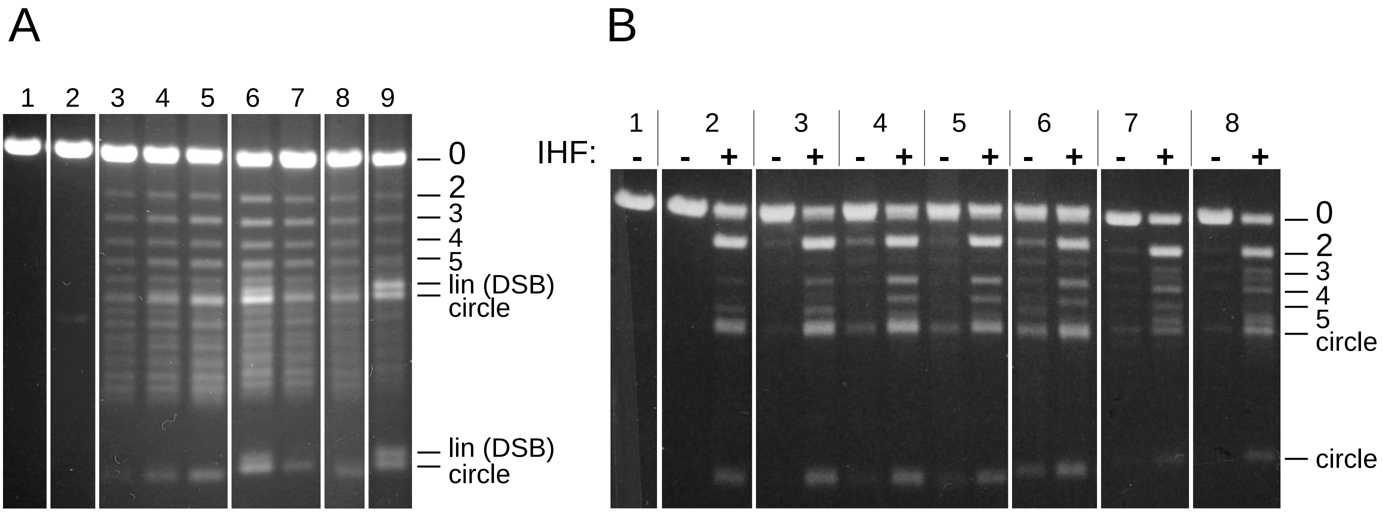
A
1 2 3 4 5 6 7 8 9
0
2
3
4
5
lin (DSB)
circle
lin (DSB)
circle
B
1 2 3 4 5 6 7 8
IHF: - - + - + - + - + - + - + - +
0
2
3
4
5
circle
circle
